# A Case of Severe Symptomatic Central Nervous System Sarcoidosis Secondary to Treatment with Adalimumab

**DOI:** 10.1155/2019/7121539

**Published:** 2019-06-16

**Authors:** Benedicta Nneoma Nnodum, Lida P. Hariri, Despoina Mavrommati, Lauren Dudley

**Affiliations:** ^1^Department of Internal Medicine, Berkshire Medical Center, Pittsfield, Massachusetts, USA; ^2^Department of Pathology, Massachusetts General Hospital, Harvard Medical School, Boston, Massachusetts, USA; ^3^Department of Rheumatology, Berkshire Medical Center, Pittsfield, Massachusetts, USA

## Abstract

Antitumor necrosis factor-*α* therapy has been used effectively in treatment of many inflammatory diseases such as rheumatoid arthritis, psoriasis, psoriatic arthritis, ankylosing spondylitis, and inflammatory bowel disease. There are increasing number of paradoxical reactions associated with this therapy that are being reported. We present the case of a 63-year-old male with psoriatic arthritis maintained on adalimumab and methotrexate (previous treatment trials of prednisone and leflunomide) who developed severe symptomatic sarcoidosis in the brain, liver, and lung. Upon discontinuation of adalimumab, the symptoms resolved but the imaging findings persisted. Although the development of sarcoidosis (usually in the lung, skin, and eyes) while on antitumor necrosis factor-*α* therapy is increasingly reported, the brain and liver are less commonly involved but should be borne in mind by physicians when extensive granulomatous lesions develop.

## 1. Introduction

Antitumor necrosis factor-*α* (TNF-*α*) therapy has been used effectively in treatment of many inflammatory diseases such as sarcoidosis, rheumatoid arthritis (RA), psoriasis, psoriatic arthritis, ankylosing spondylitis, and inflammatory bowel disease. However, these medications have been associated with several side effects that mimic the diseases they are intended to treat which hence are called paradoxical effects. Paradoxical effects are described as new onset or exacerbation of a condition that is often treated by this therapy. The most frequently reported paradoxical reactions are psoriasiform and dermatitis-like skin reactions with some studies reporting rates of more than 10% [1]. Similar manifestations include uveitis, colitis, vasculitis, and autoimmune diseases such as lupus and myositis [2]. Adalimumab is an antirheumatic, disease modifying, TNF-*α* blocking agent used in treatment of moderate to severe psoriatic arthritis and has been proven effective in multiple randomized clinical trials [3, 4]. It is usually well tolerated. The most frequently reported side effects include headache, skin rash, infection most commonly sinusitis, upper respiratory tract infection, and injection site reactions. Most of the existing safety data for adalimumab are available in patients with RA [5]. We present a patient with psoriatic arthritis maintained on adalimumab and methotrexate who developed severe symptomatic sarcoidosis in the brain, liver, and lung.

## 2. Case Description

A 63-year-old Caucasian man with a history of benign prostatic hyperplasia with urinary obstruction, distant history of motor vehicle accident status-post multiple fractures and emergency splenectomy, psoriatic arthritis (PsA), and diffuse idiopathic skeletal hyperostosis diagnosed more than 10 years ago presented with fever and weakness. His psoriatic arthritis had been initially controlled with nonsteroidal anti-inflammatory agents; however, eventually he required short courses of prednisone and methotrexate (MTX). Adalimumab was added to methotrexate when the patient was not improving. He had a sustained response to this therapy for almost 2 years. While on this combination therapy, he developed worsening joint pain, fever, left lower extremity weakness, severe myalgia in proximal thigh muscles, lower and upper extremity arthralgia, unsteady gait, and acute urinary retention. He had fever for 1 week prior to hospital admission. Physical examination upon admission was pertinent for tender bilateral, submandibular lymphadenopathy, and left lower extremity weakness (4/5 strength on the left hip flexor and 5/5 strength on the right) without meningismus, nuchal rigidity, wide-based gait without foot drop, up going toes (positive Babinski), decreased perianal sensation, and tender bilateral thighs. He needed Foley catheterization for urinary retention for four days after failing a voiding trial. 18 days prior to this hospitalization, he temporarily stopped adalimumab and methotrexate due to an active ear infection but restarted it one week prior to hospital presentation.

Other medications included atenolol, Ativan, folic acid, sumatriptan, and tamsulosin. Family history was notable for a daughter with ulcerative colitis (UC) and bile duct cancer, a son with glioblastoma, a brother with UC, and three sisters having lupus with sicca syndrome, celiac disease, and seronegative rheumatoid arthritis. He had a 25-pack year smoking history.

**Figure 1:**
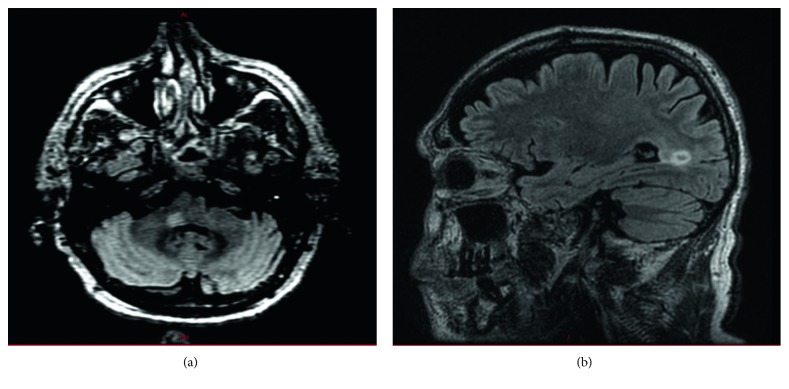
MRI of the brain showing hyperintense lesion in the right middle cerebellar peduncle lesion (a) and ring-shaped periatrial white matter lesion (b).

**Figure 2:**
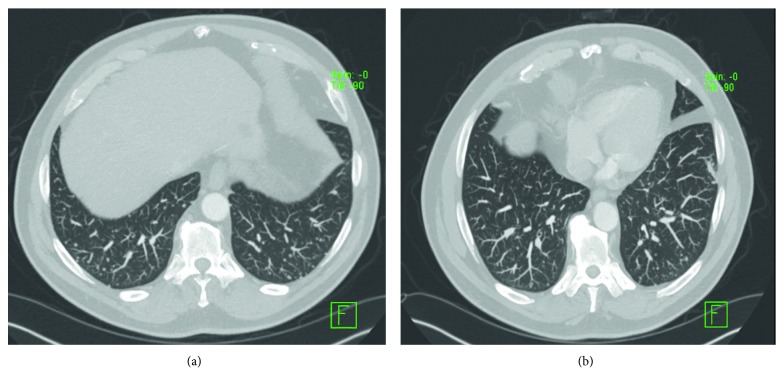
Chest CT showing numerous small lung nodules.

**Figure 3:**
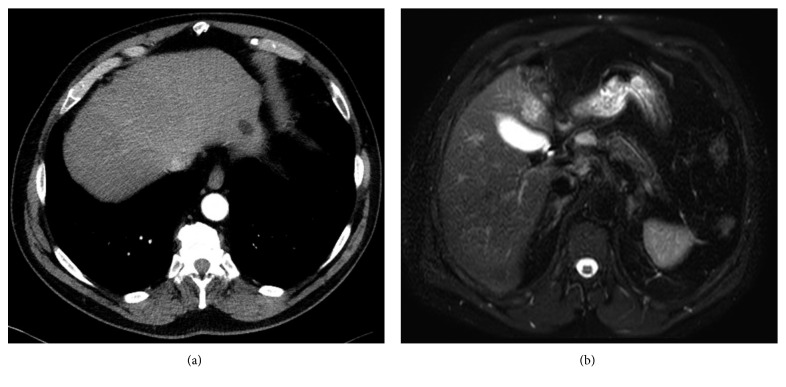
MRI abdomen showing 1.8 cm liver cyst (a) and 1.3 cm ovoid liver lesion (b).

Investigations done during the index hospitalization included brain MRI which showed T2-FLAIR hyperintense lesions in the juxtacortical, deep and periventricular white matter of the bilateral cerebral hemispheres, and infratentorial lesions in the right middle cerebellar peduncle, some with a ring-like appearance without enhancement (Figure 1). A CT scan of the chest/abdomen/pelvis demonstrated diffuse interstitial lung diease with linear opacities at the bases and numerous small nodules measuring 2-4 mm (some in clusters and some were subpleural). There were also few tree-in-bud, mediastinal, hilar, and subcarinal adenopathy with the largest measuring 1.7 cm. A 1.7 cm x 1.3 cm hypodense lesion was seen in the liver (Figure 2). MRI abdomen noted a 2.3 cm liver lesion consistent with hemangioma, 1.8 cm cyst, and a 1.3 cm ovoid lesion (Figure 3). Cerebrospinal fluid (CSF) showed cell count 101/mm^3^ (85% lymphocytes), total protein 55 mg/dl, glucose 59 mg/dl, no oligoclonal bands, JC virus polymerase chain reaction (PCR) < 500 copies, negative CSF cultures for bacteria, mycobacteria, herpes simplex virus (HSV), Epstein–Barr virus, varicella zoster virus PCR, human herpes virus-6 PCR, enterovirus, cryptococcal antigen, equivocal Lyme IgG/IgM antibody (Ab), negative Lyme western blot, galactomannan, and nonreactive venereal disease research laboratory. Blood work revealed elevated erythrocyte sedimentation rate 65 mm/hr, elevated C-reactive protein 76.2 mg/L, high normal aldolase 7.2 U/L, and normal liver function tests. Serological evaluation for infection was remarkable for equivocal Lyme ELISA but negative Lyme western blot, negative blood and gonococcal cultures, negative interferon-gamma release assay for tuberculosis, negative Babesia, malaria antigen, anaplasma, chlamydia/gonorrhea nucleic acid, negative hepatitis B core Ab, surface antigen, and hepatitis C viral Ab, nonreactive HIV, negative HSV IgM by immunofluorescence assay, cytomegalovirus (CMV) viral load, and CMV Ab. Serological evaluation for inflammatory disease was remarkable for high-titer antinuclear antibodies 1 : 640, positive scleroderma-70 Ab 41.27, high normal complement C3 level 157, positive antismooth muscle Ab- 1 : 40, normal complement C4 level 30, negative dsDNA, normal serum angiotensin converting enzyme levels, rheumatoid factor <30, negative SS-A/SS-B, and neuromyelitis optica antibodies. At this point, Neurology thought this was a rare case of drug-induced cerebral demyelination secondary to adalimumab.

He was started on ceftriaxone for possible Lyme myelitis on admission and acyclovir for possible HSV encephalitis. These medications were stopped because CSF and serum Lyme along with HSV PCR returned negative. His fever, left lower extremity weakness, gait instability, and urinary retention improved during his admission such that upon discharge; he had a normal neurologic exam and was voiding normally. He did not receive corticosteroids. He was discharged with plans to follow the central nervous system (CNS) and pulmonary and hepatic lesions as an outpatient. Subjective muscle weakness and gait instability took several weeks to completely resolve. He returned to work full-time.

**Figure 4:**
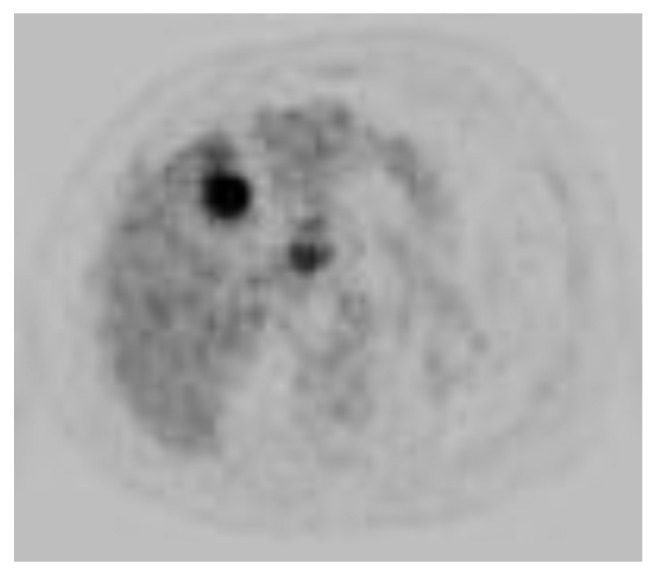
PET-CT demonstrated 3 cm hypermetabolic liver mass seen on MRI.

**Figure 5:**
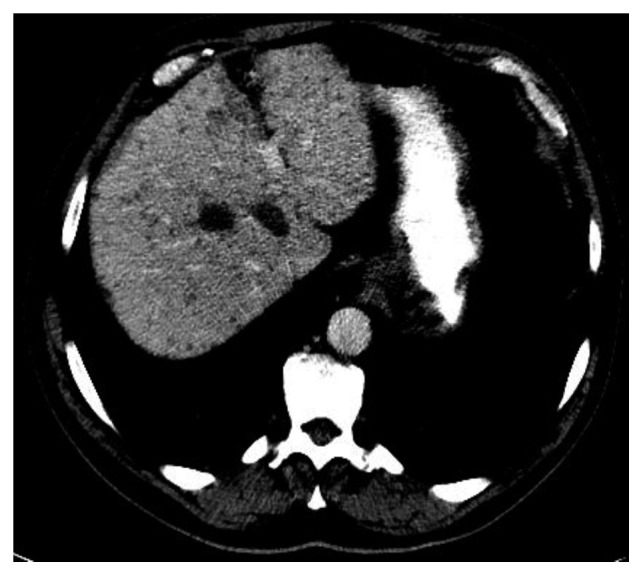
Surveillance CT abdomen/pelvis 3 months after the initial one showing numerous new tiny hepatic lesions.

**Figure 6:**
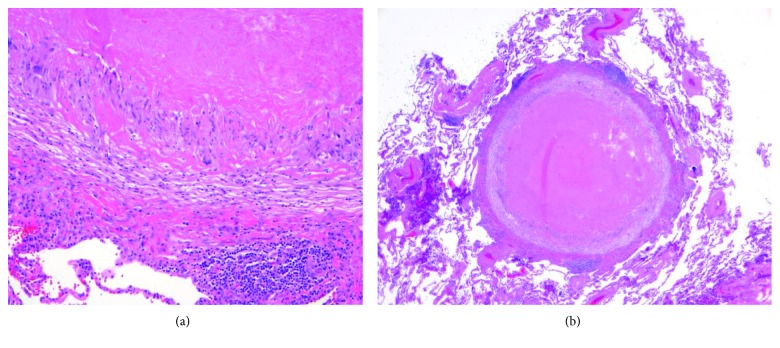
(a) Lung biopsy demonstrating caseating granuloma, magnification 20x. (b). Lung biopsy demonstrating caseating granuloma, magnification 4x.

Due to concerns raised for abnormal liver lesions, a PET-CT done after hospital discharge demonstrated a 3 cm hypermetabolic liver mass concerning for neoplasm such as hepatocellular carcinoma, sarcoid nodule, or other granulomatous lesion and a resolution of the previously noted 1.7 cm subpleural nodule (Figure 4). A subsequent liver biopsy showed septal inflammation with associated portal and lobular hepatitis which was not clearly diagnostic for a specific condition but concerning for drug-induced liver injury as anti-TNF-alpha agents have been associated with this [6]. A surveillance CT abdomen/pelvis 3 months after the initial one showed a resolving right hepatic hematoma but new numerous subcentimeter hypodense lesions throughout both hepatic lobes and numerous subcentimeter pulmonary nodules throughout both lungs (Figure 5). Concern for drug-related sarcoidosis or atypical mycobacterial infection was raised due to the multisystem involvement and drug exposure given reports in the literature [7]. A repeat PET-CT 6 months after the initial one demonstrated decreased size of the innumerable liver lesions with no new or enlarging hepatic lesions but continued numerous pulmonary nodules. He then underwent bronchoscopy followed by video-assisted thoracoscopic surgery (VATS) wedge resection, which revealed multiple caseating granulomas (Figures 6(a) and 6(b)). Cultures from the bronchoalveolar lavage (BAL) fluid yielded rare *Staphylococcus capitis* thought to be from oral flora. BAL culture remained negative for fungi, Legionella, anaerobes, and acid-fast bacilli after two months. Acid-fast bacilli and silver stain performed on tissue sections from the wedge resection were negative for organisms.

## 3. Discussion

Sarcoidosis is a granulomatous disease that may affect multiple organs; it is thought to be multifactorial in origin with environmental factors, genetic susceptibility, and microorganisms playing a role. It is diagnosed by finding noncaseating granulomas on biopsy [8] though caseating granulomas occur rarely [9]. Developing sarcoidosis during anti-TNF therapy is paradoxical as some anti-TNF agents are used in the treatment of refractory sarcoidosis [10, 11]. There are several existing hypotheses for anti-TNF induced sarcoidosis, some of which are described here. Massara et al. state that cytokine imbalance due to long term TNF-alpha suppression could lead to paradoxical reactions [12]. Cleynen and Vermeire report that TNF inhibitors may lead to excess interferon-alpha (INF-*α*) in dendritic cells [13]. The imbalance of TNF-*α* and INF-*α* can support the production of auto antigens leading to the paradoxical reactions [13]. The oldest drug responsible for this is etanercept [14]. However, due to increasingly reported cases of sarcoidosis-like lesions under other anti-TNF therapies, it is now considered a class effect [14].

There are a few case reports of development of neurosarcoidosis in patients on other TNF-alpha inhibitors such as etanercept or infliximab [15–18]. These cases are reviewed in detail in Table 1. Existing case reports of sarcoidosis-like lesions developing while on adalimumab have been described in patients with underlying diseases such as Crohn's disease and inflammatory rheumatic diseases. Almost all the cases showed noncaseating granuloma, the cornerstone of sarcoidosis. The most frequently involved organs include the lungs, skin, and eyes but sarcoidosis of the central nervous system and liver has not been described with adalimumab. In most of the cases, discontinuation of anti-TNF therapy and/or treatment with steroids led to resolution or improvement of symptoms [12]. Our patient had negative interferon-gamma release assay for tuberculosis and negative mycobacterial cultures of the cerebrospinal fluid and bronchoalveolar lavage, effectively ruling out mycobacterial infection as an explanation for his clinical presentation. This is the first published report of CNS sarcoidosis developing in the setting of adalimumab use for psoriatic arthritis.

## 4. Conclusion

The development of sarcoidosis due to anti-TNF therapy is uncommon with CNS sarcoidosis being exceedingly uncommon. Sarcoidosis should be considered when new granulomatous lesions develop in multiple organs including the CNS after excluding mycobacterial infections in patients on anti-TNF therapy.

## Figures and Tables

**Table 1:** Review of neurosarcoidosis cases in the setting of TNF-alpha inhibitor use.

Author	Anti-TNF agent used	Underlying disease	Clinical presentation	Imaging findings	Cerebrospinal (CSF) findings	Pathological findings	Outcome
Berrios et al. [15]	Etanercept	Chronic JRA and refractory uveitis	Acute mental status change, fever, headache, joint pain, dizziness, night sweats, chills	First brain MRI: diffuse abnormal hyper intensities within sulci of both cerebral hemispheres associated with abnormal leptomeningeal enhancement, 2 small nonspecific foci of fluid-attenuated inversion recovery hyperintense signals in the right thalamus and lower pons. Second brain MRI: small hyperintense cortical lesions. Cervical spine MRI: incidental abnormal lymph nodes in the right upper mediastinum. CT thorax, abdomen and pelvis: hepatosplenomegaly, lymphadenopathy in the axillary, mediastinal, intra-abdominal, iliac chain and inguinal lymph nodes. PET scan: unremarkable.	96% lymphocytes, 3% monocytes, 1% lymphocytes, protein: 48 mg/dl, glucose: 49 mg/dl.	Noncaseating granulomatous lymphadenitis	Clinical and imaging improvement.

Sturfelt et al. [16]	Infliximab, methotrexate	Erosive RA	Headache, fever, diplopia, severe papilledema of both eyes, nerve palsy of the left eye	Head CT, brain and spinal cord MRI, MR angiography: unremarkable. Transcranial Doppler analysis: high intracranial pressure.	Slight pleocytosis with mononuclear leucocytes of 49 × 10^6^/l and polynuclear leucocytes 4 × 106/l, normal CSF/blood glucose ratio, slightly increased protein of 0.78 g/l. Negative culture results for bacteria, viral, fungal or protozoan infection.	Bilateral granulomatous iridocyclitis, retinal periphlebitis	Resolution of headache, papilledema, eye muscle function. Pathological ophthalmic changes slowly subsided. Symptoms. Returned to work full-time.

Durel et al. [17]	Etanercept	Erosive RA	Bilateral facial paralysis, anosmia, papilledema, anterior uveitis, weight loss, dyspnea, sicca symptoms	Brain MRI: enhanced signal of cranial nerves. Chest CT: bilateral hilar and mediastinal lymph nodes.	Elevated protein, normal glucose without oligoclonal bands.	No histological confirmation	Complete resolution of eye sight and brain MRI findings but not facial paralysis.

Mao-Draayer et al. [18]	Adalimumab	HLA-B27-positive AS	New seizures	Head CT: hypo density in left frontal subcortical white matter. Brain MRI: leptomeningeal enhancement and several hyperintense lesions.	Mild lymphocytic leukocytosis, elevated protein.	Noncaseating granuloma	Resolution of the disease.

CNS, central nervous system; JRA, juvenile rheumatoid arthritis; RA, rheumatoid arthritis; AS, ankylosing spondylitis.
